# Trends in Amphibian Occupancy in the United States

**DOI:** 10.1371/journal.pone.0064347

**Published:** 2013-05-22

**Authors:** Michael J. Adams, David A. W. Miller, Erin Muths, Paul Stephen Corn, Evan H. Campbell Grant, Larissa L. Bailey, Gary M. Fellers, Robert N. Fisher, Walter J. Sadinski, Hardin Waddle, Susan C. Walls

**Affiliations:** 1 United States Geological Survey, Forest and Rangeland Ecosystem Science Center, Corvallis, Oregon, United States of America; 2 United States Geological Survey, Patuxent Wildlife Research Center, Laurel, Maryland, United States of America; 3 Department of Ecosystem Science and Management, Pennsylvania State University, University Park, Pennsylvania, United States of America; 4 United States Geological Survey, Fort Collins Science Center, Fort Collins, Colorado, United States of America; 5 United States Geological Survey, Northern Rocky Mountain Science Center, Missoula, Montana, United States of America; 6 United States Geological Survey, Patuxent Wildlife Research Center, Turners Falls, Massachusetts, United States of America; 7 Department of Fish, Wildlife and Conservation Biology, Colorado State University, Fort Collins, Colorado, United States of America; 8 United States Geological Survey, Western Ecological Research Center, Point Reyes Station, California, United States of America; 9 United States Geological Survey, Western Ecological Research Center, San Diego, California, United States of America; 10 United States Geological Survey, Upper Midwest Environmental Sciences Center, La Crosse, Wisconsin, United States of America; 11 United States Geological Survey, National Wetlands Research Center, Lafayette, Louisianna, United States of America; 12 United States Geological Survey, Southeast Ecological Science Center, Gainesville, Florida, United States of America; Lakehead University, Canada

## Abstract

Though a third of amphibian species worldwide are thought to be imperiled, existing assessments simply categorize extinction risk, providing little information on the rate of population losses. We conducted the first analysis of the rate of change in the probability that amphibians occupy ponds and other comparable habitat features across the United States. We found that overall occupancy by amphibians declined 3.7% annually from 2002 to 2011. Species that are Red-listed by the International Union for Conservation of Nature (IUCN) declined an average of 11.6% annually. All subsets of data examined had a declining trend including species in the IUCN Least Concern category. This analysis suggests that amphibian declines may be more widespread and severe than previously realized.

## Introduction

Amphibians have received increasing attention since a crisis of declining populations was first recognized in the late 1980s [1]–[3]. In 2004, a comprehensive global assessment of amphibian status suggested that 32.5% of the world's species and 31.7% of the United States' species were declining [4]. The current extinction rate for amphibians has been estimated to be 211 times the background rate [5]. These numbers indicate that many species have conservation problems but they do not reveal the rate of population loss. Here, we use data from the U.S. Geological Survey's Amphibian Research and Monitoring Initiative (ARMI) to estimate the rate of change in the probability that amphibians occupy ponds and other comparable habitat features across the United States.

**Figure 1:**
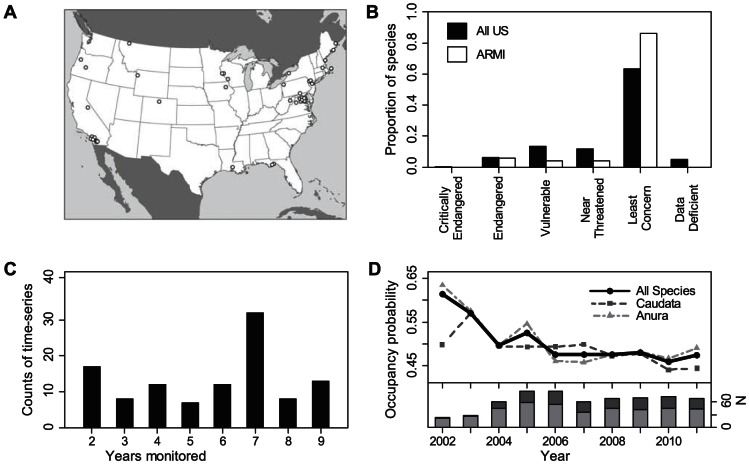
Characteristics of monitoring data. (*A*) Location of monitoring areas. (*B*) Distribution of species among IUCN categories. (*C*) Number of years monitored in each time series. (*D*) Mean annual estimates of probability of site occupancy and number of occupancy estimates (N).

Documenting the rate of change in population parameters requires intensive studies that separate true changes in populations from changes in the probability of capture or detection when amphibians are present [6]. Such studies are relatively rare and it is unusual to have sufficient trend data to assess patterns at a national scale. The occupancy estimates produced by ARMI are statistically unbiased because they use repeated surveys to account statistically for the probability of detecting a species that is present [7]. Hence, our trend estimates based on these data are not influenced by changes in detection, though they rely on data points that each have associated error. Each occupancy estimate that we analyze applies to a species at a study area and each study area has a range of inference spanning tens to hundreds of sites. For heuristic purposes, the probability of site occupancy can be thought of as the expected proportion of sites occupied within the study area [7]. These occupancy estimates span a broad range of habitats, geographic areas (Figure 1A), and species including International Union for Conservation of Nature (IUCN) categories ranging from Endangered to Least Concern (Figure 1B).

Previous large analyses of amphibian time series relied on count data from individual populations [8]–[10]. We present the first broad assessment of amphibian trends to conform with a recommendation to document change in the number of populations rather than change in abundance [11].

## Methods

We analyzed estimates of occupancy available at armi.usgs.gov. Each study within ARMI that generated these estimates used some form of repeated observation to detect amphibians [12]–[16]. An observation was usually a visual encounter survey but trapping and calling surveys were sometimes used for logistical reasons or to increase detection probability. Repeated observations were then used to estimate the proportion of sites where a species was present while accounting for imperfect detection [17]. Because the probability of detecting a species that is present is estimated and accounted for in each occupancy estimate, methods need not be standardized across studies and any changes in detection probability over time will not bias trend estimates.

A site was a pond, watershed, plot, or for calling surveys, was the area within hearing distance of a point-survey location. A study area was the range of inference for a set of sites. Each study encompassed a variable number of sites that were monitored for the presence of target species. Multiple species of amphibian were monitored at many of the study areas. Each study generated annual estimates of occupancy using either a single-season occupancy estimator [17] or a multi-season dynamic occupancy model [18]. In the latter case, a form of model was used that estimates occupancy each year without imposing trends. We analyzed all time-series with two or more consecutive annual occupancy estimates (Figure 1C).

For our analysis of these occupancy estimates, we used generalized-linear mixed models to estimate mean occupancy each year and mean trends in occupancy for each time series of occupancy estimates. We fit models using the lme4 package [19] in the R programming language [20]. All models used a similar random effects structure with an among-time-series random effect to account for variation in mean occupancy (random intercept) and an among-time-series random effect for factors describing among year differences (random slope). Occupancy estimates were weighted by the inverse of their variance derived from their standard error. We replaced standard errors <0.04 with 0.04 so that no single occupancy estimate would be given disproportionate weight and to account for cases where standard errors were estimated poorly due to occupancy being close to 0 or 1.

To estimate mean occupancy each year, we treated year as a factor. To estimate mean trends over years, we treated year as a continuous covariate where year was standardized to have a mean of 0 for each time series. To compare trends among subsets of the data, we included a fixed effect for one of several grouping variables (IUCN category, taxon, geography, management agency). We allowed differences among groups in both the mean occupancy and the mean trend in occupancy across years. Models were run using a log-link function to estimate relative rates of change in occupancy over years. We report the annual rate of change which is e*^β^*-1 where *β* is the instantaneous rate of change from the log-linear models. We used the delta method to obtain the SE for the annual rate of change. For comparison, we also ran models using an identity link to estimate absolute instantaneous changes in occupancy. We used likelihood-ratio tests (LRT) to evaluate the null hypothesis of no difference in trend among subsets of the data indicated by the grouping variables.

## Results

**Figure 2:**
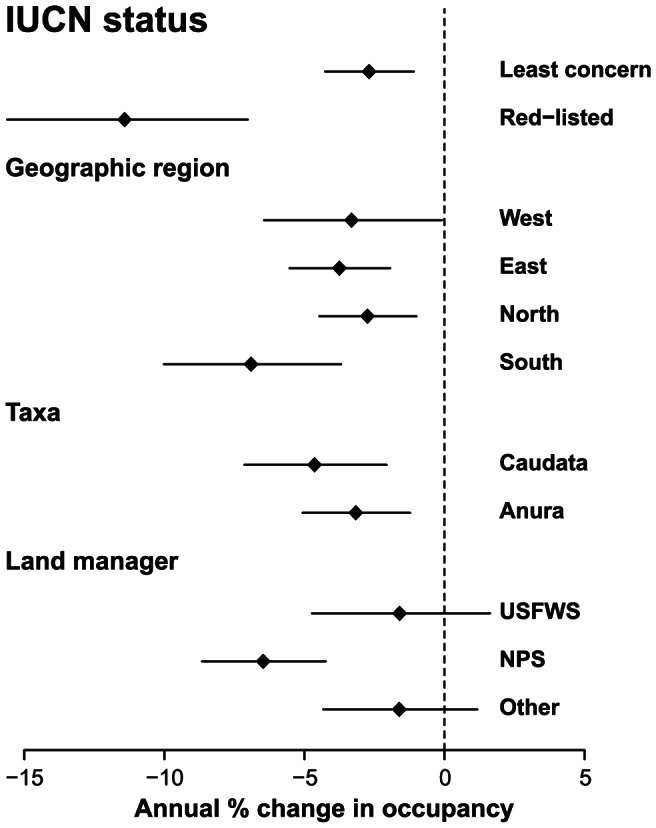
Rate of change in the probability of site occupancy for subsets data. “Red-listed” includes species that the IUCN categorizes as Near Threatened, Vulnerable, or Endangered. The geographic regions of the United States are overlapping and are North or South of 39° latitude or East or West of −104° longitude. Major land managers include the U.S. Fish and Wildlife Service (USFWS) and the National Park Service (NPS). Plotted values are means and standard errors.

From 2002 to 2011, ARMI generated 612 estimates of the probability of site occupancy for 108 time series (range 2 to 9 years, Figure 1C), including 45 species and 3 species complexes at 34 study areas. Mean annual estimates of occupancy generally decreased (Figure 1D), changing at a rate of −3.7% (SE = 1.5) annually across all time series (N = 108). All subsets of data that we examined showed a declining trend (Figure 2). The time series for species categorized as Least Concern by the IUCN (N = 96) had a mean annual trend of −2.7% (SE = 1.6), while time series for species in the Endangered, Vulnerable, and Near Threatened categories (N = 12) had a mean annual trend of −11.6% (SE = 4.3). Although the number of imperiled species is highest in the western U.S. [4], [21], we did not find geographic differences in the rate of change in occupancy (LRT, 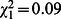, p = 0.906 for East vs. West; LRT, 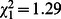, p = 0.256 for North vs. South). We also did not find convincing differences between anurans and caudates (LRT, 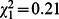, p = 0.644) or on lands managed by different agencies (LRT, 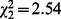, p = 0.280). Conclusions did not differ when linear rather than log-linear models were fit (Table S1). We estimated trends for individual species using a separate model that treats species as a random effect (Table S2). However, data were sparse for most species and the strength of our analysis comes from examining mean trends across a large set of species and areas.

## Discussion

Statistically unbiased estimates of the rate of change in amphibian patch occupancy are necessary to understand the scale and severity of amphibian losses [8], . They are particularly useful for species considered to be of Least Concern whose trends may be more subtle than for species determined to be imperiled at some level by the IUCN. An average loss of 2.7% of occupied sites each year for the species of Least Concern monitored by ARMI is alarming given that these species are thought to be relatively unaffected by global amphibian declines. This finding suggests that the IUCN threat status has been underestimated for some of these species. This is not a criticism of the IUCN effort, but illustrates the added value of statistically robust monitoring data to inform managers and policy makers.

Sites sampled by ARMI were designed to be roughly equivalent to populations but the relationship between sites and populations is variable and not precisely known. We characterize our rate estimates as addressing change in the occupancy of habitat patches but in one study area the scale was small watersheds with an average of 8.6 ponds in each. Trends in occupancy should not be equated with trends in density [22]. Occupancy studies necessarily include occupied and unoccupied patches. Therefore, trends in occupancy reflect a process involving both local extinctions at occupied patches and colonization of unoccupied patches [18].

Primary hypotheses to explain global amphibian declines are land use change, disease, global climate change, and interactions of these factors with each other or with other stressors like contaminants or habitat degradation [23]. Anthropogenic habitat loss is rare at ARMI study areas. The fungal pathogen associated with chytridiomycosis is found throughout the US and is common in most [24], [25] but not all [26] ARMI study areas where tested. Presence of the fungus resulted in reduced survival of adult amphibians in one study [27], but it is difficult to establish a direct link to declines in occupancy. Major die offs of amphibians were not observed in any of the studies analyzed here. The role of climate in changes in occupancy is difficult to evaluate for relatively short time series and we expect that patterns in occupancy caused by climate change will take years to become evident. The decade during which ARMI collected data experienced severe, but not unprecedented, drought [28]. Because most of the amphibians monitored rely on the presence of water for reproduction and development, precipitation patterns are an obvious hypothesis to explain changes in occupancy [16]. The relationship between occupancy trends and any potential driver is likely to vary across regions, habitats, and species necessitating careful specification of mechanisms prior to analysis of drivers.

Because the species and areas that ARMI monitors are not random, the declines we documented cannot be extrapolated directly to the rest of the U.S. or worldwide. This caveat also applies to all existing compilations of trends in amphibian abundance [8],[9]. However, it is useful to consider how our trend estimates may compare to the larger population of species and areas in the U.S, which in most cases have larger distributions than our monitoring areas (armi.usgs.gov/national_amphibian_atlas.php). The species and areas monitored by ARMI were generally selected to evaluate the status and trends of amphibians on federally-managed lands at the scale of management units [29]. Such lands are sometimes perceived as better protected than private lands. In many cases, monitoring areas were selected to target a specific imperiled species but, by design, other local species were also monitored. Hence, our analysis includes a broad range of species that span most IUCN categories of endangerment (Figure 1B), but Least Concern species are overrepresented (86% compared to 63% nationally). Also, the first year of occupancy estimates was 2002, long after many severe declines are thought to have begun [9], [30]–[32]. These factors are evidence that our analysis may underestimate the actual rate of amphibian losses in the United States. However, we emphasize that the true direction and magnitude of sampling bias is unknown and the relatively short time period monitored may not be representative of longer trends. We also note that our estimates of trends are based on estimates of occupancy that each have associated error (see armi.usgs.gov for SEs). Nonetheless, the trends we found represent the only broad assessment of population losses for amphibians in the U.S.

**Figure 3:**
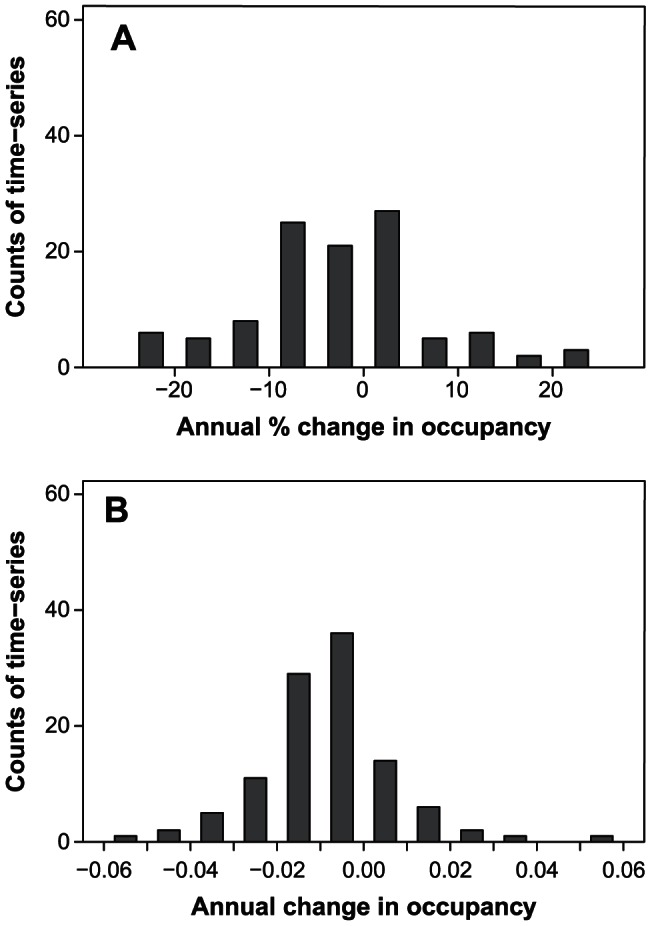
The distribution of trend estimates. Data are trends in the probability of site occupancy based on (*A*) log-linear and (*B*) linear models.

There is more than one way to estimate trends in occupancy estimates. We used log-linear models to estimate occupancy in a given year as a proportion of the previous year's occupancy. A change from 0.5 to 0.25 and a change from 0.1 to 0.05 both represent a 50% decline though the latter might involve a change in the occupancy status of a small number of sites relative to the former. As a result, similar absolute changes in occupancy influence estimates of trend more for areas where occupancy is low than for areas where occupancy is high. An alternative approach is to use linear models that estimate absolute changes in occupancy. Both methods are valid, but their sensitivity to extreme occupancy estimates and the interpretation of their estimates differ. For example, both methods suggest declines in all subsets of data examined (Table S1), but the distribution of trend estimates produced by log-linear models has a greater number of extreme negative estimates (Figure 3).

## Conclusions

We provide a synthesis of a monitoring program that is unique in its national scope and use of statistically unbiased occupancy estimates. Our trend estimates are consistent with other analyses showing that amphibians are declining [4], [8], [9], and go further by suggesting that species for which there has been little conservation concern or assessment focus (e.g., common species) may also be declining. While there was some variation across the U.S., the trend was consistently negative. Furthermore, declines are occurring on lands managed by federal agencies with the greatest observed rate of decline on National Park Service lands where management policy prescribes protection of natural ecosystem processes. Overall, the trends we documented suggest that amphibian declines may be more widespread and severe than previously thought.

## Supporting Information

Table S1 **Comparison of instantaneous trend estimates derived from linear and log-linear models of change in amphibian occupancy at ARMI monitoring areas, 2002–2011.**
(DOC)

Table S2 **Trends in the probability of site occupancy by species for ARMI monitoring areas, 2002–2011.** The estimated trend effects are annual proportional changes in occupancy and are conditional on a random effect for a variable coding species that was included in the statistical model. Caution should be taken in interpreting results for individual species as data are generally sparse. The strength of our study comes from making inferences across a broad set of species and areas.
(DOCX)
