# Supplementary material for: Trends in Amphibian Occupancy in the United States
Source: PLoS One. 2013 May 22;8(5):e64347. doi: 10.1371/journal.pone.0064347 (PMC3661441; doi:10.1371/journal.pone.0064347)
Supplement: Table S1 — Comparison of instantaneous trend estimates derived from linear and log-linear models of change in amphibian occupancy at ARMI monitoring areas, 2002–2011. (DOC) [file pone.0064347.s001.doc]

**Table S1. Comparison of instantaneous trend estimates derived from linear and log-linear models of change in amphibian occupancy at ARMI monitoring areas, 2002-2011**.

|  | log-linear trend (relative) | | | linear trend (absolute) | | | n | | |
| --- | --- | --- | --- | --- | --- | --- | --- | --- | --- |
|  | β_1_^$^ | SE | p | β_1_ | SE | p | time series | areas | species* |
| All time series | -0.038 | 0.016 |  | -0.009 | 0.00021 |  | 108 | 34 | 48 |
|  |  |  |  |  |  |  |  |  |  |
| IUCN Category |  |  |  |  |  |  |  |  |  |
| Least Concern | -0.027 | 0.016 | 0.070 | -0.0073 | 0.00022 | 0.225 | 96 | 31 | 41 |
| Red-listed | -0.124 | 0.049 |  | -0.0211 | 0.00062 |  | 12 | 9 | 7 |
|  |  |  |  |  |  |  |  |  |  |
| Taxa |  |  |  |  |  |  |  |  |  |
| Caudata | -0.048 | 0.027 | 0.644 | -0.0052 | 0.00036 | 0.468 | 37 | 23 | 14 |
| Anura | -0.032 | 0.020 |  | -0.0107 | 0.00026 |  | 71 | 29 | 34 |
|  |  |  |  |  |  |  |  |  |  |
| Land Manager |  |  |  |  |  |  |  |  |  |
| USFWS | -0.016 | 0.032 | 0.280 | -0.0051 | 0.00041 | 0.828 | 23 | 12 | 9 |
| NPS | -0.067 | 0.024 |  | -0.0103 | 0.00031 |  | 43 | 13 | 22 |
| Other | -0.016 | 0.028 |  | -0.0102 | 0.00041 |  | 42 | 9 | 30 |
|  |  |  |  |  |  |  |  |  |  |
| Region |  |  |  |  |  |  |  |  |  |
| West | -0.034 | 0.033 | 0.906 | -0.0115 | 0.00039 | 0.654 | 28 | 9 | 15 |
| East | -0.038 | 0.019 |  | -0.0080 | 0.00025 |  | 80 | 25 | 33 |
|  |  |  |  |  |  |  |  |  |  |
| North | -0.028 | 0.018 | 0.256 | -0.0072 | 0.00024 | 0.475 | 87 | 27 | 33 |
| South | -0.072 | 0.034 |  | -0.0132 | 0.00041 |  | 21 | 7 | 19 |

*45 species plus 3 species complexes.

^$^ β_1_ is the instantaneous rate of change in the log-linear model. Annual changes in the proportion of occupied sites are given by .
