# Supplementary material for: Trends in Amphibian Occupancy in the United States
Source: PLoS One. 2013 May 22;8(5):e64347. doi: 10.1371/journal.pone.0064347 (PMC3661441; doi:10.1371/journal.pone.0064347)
Supplement: Table S2 — Trends in the probability of site occupancy by species for ARMI monitoring areas, 2002–2011. The estimated trend effects are annual proportional changes in occupancy and are conditional on a random effect for a variable coding species that was included in the statistical model. Caution should be taken in interpreting results for individual species as data are generally sparse. The strength of our study comes from making inferences across a broad set of species and areas. (DOCX) [file pone.0064347.s002.docx]

**Table S2. Trends in the probability of site occupancy by species for ARMI monitoring areas, 2002-2011. The estimated trend effects are annual proportional changes in occupancy and are conditional on a random effect for a variable coding species that was included in the statistical model.  Caution should be taken in interpreting results for individual species as data are generally sparse. The strength of our study comes from making inferences across a broad set of species and areas.**

| Genus | Species | Years^*^ | Areas^$^ | Trend |
| --- | --- | --- | --- | --- |
| Plethodon | shenandoah | 4 | 1 | -0.207 |
| Hyla | versicolor | 6 | 2 | -0.174 |
| Ambystoma | tigrinum | 12 | 2 | -0.167 |
| Pseudacris | triseriata/maculata | 6 | 2 | -0.163 |
| Lithobates | pipiens | 6 | 2 | -0.120 |
| Lithobates | sphenocephalus | 16 | 2 | -0.116 |
| Lithobates | grylio | 3 | 1 | -0.114 |
| Lithobates | palustris | 7 | 1 | -0.113 |
| Anaxyrus | californicus | 18 | 3 | -0.112 |
| Bufo | americanus/fowleri | 7 | 1 | -0.103 |
| Plethodon | cinereus | 4 | 1 | -0.095 |
| Hyla | versicolor/chrysoscelis | 7 | 1 | -0.090 |
| Ambystoma | talpoideum | 3 | 1 | -0.079 |
| Pseudacris | sierra | 5 | 1 | -0.070 |
| Rana | sierrae | 5 | 1 | -0.064 |
| Anaxyrus | fowleri | 9 | 1 | -0.061 |
| Pseudacris | crucifer | 26 | 5 | -0.057 |
| Rana | pretiosa | 2 | 1 | -0.049 |
| Pseudacris | fouquettei | 9 | 1 | -0.044 |
| Notophthalmus | viridescens | 7 | 1 | -0.042 |
| Hyla | chrysoscelis | 9 | 1 | -0.040 |
| Lithobates | septentrionalis | 4 | 1 | -0.036 |
| Rana | cascadae | 2 | 1 | -0.035 |
| Rana | luteiventris | 13 | 2 | -0.031 |
| Lithobates | sylvaticus | 97 | 17 | -0.028 |
| Incilius | nebulifer | 9 | 1 | -0.026 |
| Pseudacris | ornata | 3 | 1 | -0.020 |
| Ambystoma | opacum | 7 | 1 | -0.020 |
| Taricha | granulosa | 10 | 2 | -0.017 |
| Ambystoma | macrodactylum | 16 | 3 | -0.016 |
| Ambystoma | maculatum | 77 | 13 | -0.014 |
| Eurycea | bislineata | 17 | 3 | -0.013 |
| Ambystoma | gracile | 10 | 2 | -0.009 |
| Rana | aurora | 8 | 1 | -0.003 |
| Hyla | squirella | 9 | 1 | -0.002 |
| Pseudacris | regilla | 10 | 2 | 0.001 |
| Desmognathus | fuscus | 17 | 3 | 0.009 |
| Lithobates | clamitans | 22 | 4 | 0.012 |
| Anaxyrus | canorus | 5 | 1 | 0.013 |
| Hemidactylium | scutatum | 7 | 1 | 0.022 |
| Lithobates | catesbeianus | 16 | 2 | 0.028 |
| Pseudotriton | ruber | 13 | 3 | 0.033 |
| Bufo | americanus | 6 | 2 | 0.034 |
| Hyla | cinerea | 9 | 1 | 0.043 |
| Pseudacris | maculata | 15 | 2 | 0.048 |
| Anaxyrus | boreas | 21 | 4 | 0.078 |
| Gastrophryne | carolinensis | 9 | 1 | 0.081 |
| Acris | blanchardi | 9 | 1 | 0.161 |

^*^ The total number of years across all time series

^$^ The total number of monitoring areas that include each species
